# Supplementary material for: Are primary school children attending full-day school still engaged in sports clubs?
Source: PLoS One. 2019 Nov 22;14(11):e0225220. doi: 10.1371/journal.pone.0225220 (PMC6874300; doi:10.1371/journal.pone.0225220)
Supplement: Attachment — Submitted filename: The study by Sarah Spengler explored whether primary school children attending full.docx [file pone.0225220.s002.docx]

The study by Sarah Spengler explored whether primary school children attending full-day schools still engaged in sports clubs. The topic is interesting and seems important in Germany, as number of children attending full-day schools has increased with high speed. However, there are some consideration as follows;

(1) The conception of “sports club” is ambiguous. Does it mean all kinds of sports club or out-school sports club? It seems that only out school sports club is the object, but it needs to be clarified in study purpose.

(2) Why is out-school sports club so important for children’s early development? Besides a few sentences in introduction, the lack of statistical analysis and in-depth discussion made the argument unconvinced to readers.

(3) There were too more unnecessary descriptions in Introduction and Discussion, but not enough content of results. I suggest the authors 1) reword the Introduction carefully, conclude the past studies with highly condensed sentences and directly bring up the importance of the present study. 2) make more statistical analysis to expand the present results. 3) try to avoid duplications of results in Discussion, state the principle finding of the study only, make more in-depth discussion about how the present study relates to past studies and how the present study adds new views to the thesis.

For example, the great length is used to indicate those missing numbers of each LS7 component from line 1 to line 17 on page 9. These should be detailed using a flow chat.

(4) The symbol within numbers should be “,” instead of “.”.
