# Supplementary material for: Are primary school children attending full-day school still engaged in sports clubs?
Source: PLoS One. 2019 Nov 22;14(11):e0225220. doi: 10.1371/journal.pone.0225220 (PMC6874300; doi:10.1371/journal.pone.0225220)
Supplement: Attachment — Submitted filename: REVIEW MANUSCRIPT Are primary school children attending full-day school still engaged in sports clubs.docx [file pone.0225220.s003.docx]

REVIEW MANUCRIPT

**Title: Are primary school children attending full-day school still engaged in sports clubs?**

1. **Introduction.** Comprehensive

2. **Methods**

*-a) Sample*. Not respresentative of the schoolds in the city (44% schools accepted). Please comment.

b) *Use of MoMo questionnaire* to assess sport’s club participatio:

This questionnaire was validated at older ages, than the ones studied in the manucript.

Authors shoud comment on this aspect, as studying younger ages may compromise the quality of the report.

On the other hand, the validity found fro this questionnaire is low (0.33)

2. **Results**
a) Please indicate what d you refer as “younger ages”
b) No indication of the time of physical activity within the school (number of hours of Physical Education classes), which could be diffrent given socio-economic status.
c) Nor indication either of time in games in recess or free time.
3. **Other aspects**

Phrases should not be initiated with a preposition (for, then, or …etc).
